# Need to improve availability of “access” group antibiotics and reduce the use of “watch” group antibiotics in India for optimum use of antibiotics to contain antimicrobial resistance

**DOI:** 10.1186/s40545-019-0182-1

**Published:** 2019-07-17

**Authors:** Sumanth Gandra, Anita Kotwani

**Affiliations:** 1 Department of Medicine, Washington University School of Medicine, Saint Louis, MO USA; 2 Department of Pharmacology, V. P. Chest Institute, University of Delhi, Delhi, 110007 India

Antimicrobial resistance (AMR) is a growing public health threat and one of its biggest drivers is inappropriate use of antibiotics [1]. To optimize the use of antimicrobials, the World Health Organization (WHO) in 2017 updated the Essential Medicine List (EML) and categorized the antibiotics into three groups- Access, Watch and Reserve (AWaRe) [2]. The Access group antibiotics are the first and second choices for empirical treatment of 21 common clinical syndromes. First choices are narrow spectrum agents whereas second choice are broad spectrum agents with higher resistance potential. Antibiotics such as amoxicillin/ampicillin, benzathine penicillin, trimethoprim-sulfamethoxazole, amoxicillin-clavulanic acid, cloxacillin are first choice agents. Watch group includes antibiotics with high resistance potential when compared with the Access group and includes antibiotics such as third generation cephalosporins, fluoroquinolones and carbapenems. The Reserve group includes antibiotics of last-resort like polymyxins, fourth and fifth generation cephalosporins. WHO recommends that Access group of antibiotics should be widely available and at an affordable cost and minimize the use of other two groups of antibiotics. A recent report analyzed antibiotic sales data in India between 2007 and 2012 which showed that the consumption of Watch group and Reserve group antibiotics are increasing rapidly when compared to Access group of antibiotics [3]. Here we discuss the consumption patterns of certain antibiotics in Access group and Watch group and the reasons for their consumption patterns.

Human antibiotic consumption in India is among the highest in the world [4]. Antibiotic consumption in India increased from 3.2 billion DDDs (defined daily doses) in 2000 to 6.5 billion DDDs in 2015 [4]. Although per-capita consumption of antibiotics in India is low when compared to several other countries [4], the proportion of broad-spectrum antibiotics (Watch group and Reserve group) consumption is high as evident in analysis of pediatric antibiotic sales data in 70 countries [5]. The use of third generation cephalosporins which belongs to Watch group is increasing rapidly in India [6]. In contrast to the rapid rise in the consumption of third generation cephalosporins between 2000 and 2015, the consumption of fluoroquinolones (Watch group antibiotics) is decreasing whereas penicillins (Access group antibiotics) consumption remained constant [6].

The combination of broad-spectrum activity, availability in oral and parenteral formulations, convenient dosing and minimal adverse effects make second and third generation cephalosporins an ideal choice for prescribers in both adults and children. Although, second and third generation cephalosporins offer several advantages, their use increases the risk of colonization with extended spectrum beta-lactamase (ESBLs) producing Enterobacteriaceae (e.g.: *E. coli*) that are part of human normal intestinal flora [7, 8]. The high prevalence of third generation cephalosporin resistant Enterobacteriaceae among clinical isolates obtained from both children and adults in India [9] correlates well with the increasing consumption of third generation cephalosporins due to selection pressure. In a national scale study 2014, using a large private diagnostic laboratory network data, approximately 80% of the *E.coli* isolates obtained from blood cultures were resistant to third generation cephalosporins [9].

The increasing use of second and third generation cephalosporins belonging to Watch group could be attributed to combination of factors such as changing prescribing practices, increasing antimicrobial resistance to other antibiotic classes and lack of availability of first-line penicillin antibiotics belonging to Access group.

## Changing prescribing practices

In a 2004 study assessing antibiotic consumption in private retail pharmacies in Delhi showed that fluoroquinolones were most commonly consumed antibiotics followed by penicillins and cephalosporins [10]. Consumption of cephalosporins was about 39% less than fluoroquinolone. A repeat study in 2007 in the same areas in private retail pharmacies showed equal consumption of cephalosporins and fluoroquinolones [11]. Three most commonly prescribed cephalosporins were cefuroxime, cefixime and combination of cefixime and clavulanic acid all of which belonged to Watch group [11].Penicillins are recommended as the first line antibiotics for treatment of respiratory tract infections [12]. However, recent trends indicate that penicillins are increasingly substituted by second and third generation cephalosporins for treatment of respiratory tract infections in both outpatient [13, 14] and inpatient settings [15, 16]. In a study between December 2007 and November 2008 which conducted exit interviews of patients with upper respiratory tract infections showed that in private clinics, cephalosporins were prescribed to 39% of patients followed by fluoroquinolone (24%), penicillins (19%) and macrolides (11%) [13]. However, in public clinics, the main class of antibiotics prescribed were penicillins (31%), followed by macrolides (25%), fluoroquinolones (20%) and cephalosporins (10%). In a 2014 qualitative study involving focus-group discussions and face-to-face interviews with doctors and pharmacists to study the behavior and perception about antibiotic use for acute upper respiratory tract infections (URTIs) and diarrhea in children in New Delhi indicated that some General Practitioners use cefixime and cefpodoxime for URTIs [14]. In a retrospective five-year study between 2007 and 2012 among hospitalized patients with community acquired pneumonia showed that cephalosporin prescriptions significantly increased whereas penicillin prescriptions significantly decreased over the study period [15]. Among the total antibiotic prescriptions, cephalosporin consumption increased from 16% in 2007 to 27% in 2012, whereas penicillins consumption decreased from 30% in 2007 to 16% in 2012 [15]. In a recent multicenter study involving six pediatric hospitals in 2016, third generation cephalosporins were the most commonly prescribed antibiotics for lower respiratory tract infections (LRTIs) and accounted for 37% of the prescriptions for LRTIs among hospitalized children [16].

Changing prescribing practices among healthcare providers could be due to multiple factors and one such factor could be the influence of marketing and promotion practices of drug manufactures. Although third generation cephalosporins belonging to Watch group are increasingly used for treatment of respiratory tract infections, studies indicate that penicillins belonging to Access group are still active against the common bacterial pathogens causing respiratory tract infections. In a recent study examining resistance rates of pathogens isolated from community acquired pneumonia patients in India between 2012 and 2014, showed high susceptibility to amoxicillin/ampicillin and amoxicillin-clavulanic acid [17]. In this study, the susceptibility rates of *Streptococcus pneumoniae* and *Streptococcus pyogenes* for amoxicillin were 92 and 100%, respectively. For *Hemophilus influenzae*, ampicillin susceptibility was 91% and amoxicillin/clavulanic acid was 97%, respectively [17]. These findings reassure prescribers to follow the published treatment guidelines by National Center for Disease Control and INDIACLEN Taskforce on Pneumonia, which recommend use of Access group of antibiotics (amoxicillin, amoxicillin-clavulanic acid and ampicillin-gentamicin (in young children)) for community acquired respiratory tract infections as empiric treatment choice in both outpatient and inpatient settings [18, 19]. Third generation cephalosporins belonging to Watch group should be reserved for very sick patients or patients admitted to intensive care units or when there is a clinical deterioration on first line agents belonging to Access group.

## Increasing antimicrobial resistance to fluoroquinolones

Quinolones belonging to Watch group have been the main stay of treatment for enteric fever and bacterial dysentery which account for a huge burden of infections in India. Several recent studies reported increasing quinolone resistance among bacteria causing these infections. In a national scale retrospective study on antimicrobial susceptibility of various bacterial pathogens between 2008 and 2014, *Salmonella* Typhi resistance to nalidixic acid was 98% [9]. Similarly, ciprofloxacin resistance among *S*. Typhi was 69% in 2014 as per Indian Council of Medical Research (ICMR) AMR surveillance network hospitals [20]. Quinolone resistance among *Shigella spp*. and *Campylobacter spp*. causing bacterial dysentery is also increasing. In a recent study from South India showed that *Shigella flexneri* and *S. sonnei* resistance to nalidixic acid was greater than 95% [21]. Similarly, in a study between 2008 and 2010 assessing the *Campylobacter spp.* susceptibility patterns showed that 100% of the isolates were resistant to nalidixic acid and ciprofloxacin [22]. Increasing quinolone resistance has resulted in the use of third generation cephalosporins (ceftriaxone and cefixime) belonging to Watch group as empiric treatment choices for enteric fever and bacterial dysentery. However, recent data indicate that *S.* Typhi has become susceptible to older antibiotics like amoxicillin, trimethoprim/sulfamethoxazole and chloramphenicol which belong to Access group. In a national scale study and 2014 ICMR AMR surveillance data, 95% of *S*. Typhi isolates in 2014 were susceptible to ampicillin, chloramphenicol and trimethoprim/sulfamethoxazole [9, 20]. These results indicate the opportunity to examine and report outcomes using older first-line antibiotics (amoxicillin, trimethoprim/sulfamethoxazole and chloramphenicol) belonging to Access group among mild and moderately severe enteric fever cases especially in centers where microbiological and antimicrobial susceptibility services are available.

## Lack of availability of narrow-spectrum antibiotics

The use of Watch group antibiotics namely second and third generation cephalosporins is associated with widespread availability of these antibiotics. A review of the April–July 2017 edition of the Current Index of Medical Specialties (CIMS) India, showed that 135 pharmaceutical companies were manufacturing cefixime (third-generation cephalosporin), 103 were manufacturing cefpodoxime (third generation cephalosporins) and 69 were manufacturing cefuroxime (second generation cephalosporin) [6]. In contrast, 112 companies were manufacturing amoxicillin-clavulanic acid, 51 were manufacturing amoxicillin and only one company was manufacturing penicillin and benzathine penicillin [6]. The lack of availability of Access group antibiotics such as amoxicillin and benzathine penicillin was evident in studies focused on availability of antibiotics in public and private pharmacies. A study was conducted in New Delhi (July–October 2011) to examine the access of antibiotics at primary care, secondary care and tertiary care facilities of public health providers in Delhi and private retail pharmacies [23]. The results indicated that benzathine penicillin was not available at primary care, secondary care and tertiary care facilities of public sector surveyed, whereas ampicillin suspension was available only in 25% of primary care facilities and was not available in secondary and tertiary care facilities. However, cefuroxime which is part of Delhi Government’s hospital Essential Medicine List (EML)but not for primary care, was available at 44% of primary care facilities. Cefixime which was not part of Delhi Governments’ EML was available in 22% of primary care facilities and 14% of secondary care facilities. Similar to public sector, benzathine penicillin was not available in any private retail pharmacies however amoxicillin and cefixime were available in greater than 90% of the private pharmacies [23]. Interestingly, the median price ratio (MPR) of second and third generation cephalosporins belonging to Watch group is lower when compared to amoxicillin belonging to Access group in both public sector procurement agencies and private retail pharmacies [23].The MPR is the local median unit price of a medicine in comparison with the median unit price of international reference price which was found in the Management Sciences for Health (MSH) Price Indicator Guide.

## Conclusions

The current situation in India shows that there is excess use and availability of Watch group of antibiotics compared to Access group of antibiotics. Indian authorities should consider revising the National EML and adopt the WHO EML for antibiotics in three categories (AWaRe) to optimize the use of antimicrobials. Measures should be taken by regulators and policy makers to improve the availability and use of Access group of antibiotics and at the same time decrease the use of Watch group of antibiotics that have higher resistance potential in both public and private sector. A policy decision can be taken to ask at least public sector pharmaceutical manufacturing units to manufacture Access group of antibiotics and these antibiotics should be available at government owned ‘Generic Drug’ stores and procured for all public sectors. Standard treatment guidelines should be prepared based on current sensitivity patterns and implemented at public and private facilities so that doctors start prescribing Access group of antibiotics as first line drugs. Prescription audits at all level of healthcare and feedback to prescribers and facility manager will further improve the quality of prescribing.

## Abbreviations

CIMS: Current index of medical specialties
DDD: Defined daily dose
*E coli*: *Escherichia coli*
EML: Essential medicine list
ESBL: Extended spectrum beta-lactamase
INR: Indian rupee
LRTIs: Lower respiratory tract infections
*S. Typhi*: Salmonella Typhi
URTIs: Upper respiratory tract infections
WHO: World health organization
